# The Woody Planet: From Past Triumph to Manmade Decline

**DOI:** 10.3390/plants9111593

**Published:** 2020-11-17

**Authors:** Laurence Fazan, Yi-Gang Song, Gregor Kozlowski

**Affiliations:** 1 Department of Biology and Botanical Garden, University of Fribourg, Chemin du Musée 10, 1700 Fribourg, Switzerland; laurence.fazan@unifr.ch; 2 Eastern China Conservation Center for Wild Endangered Plant Resources, Shanghai Chenshan Botanical Garden, Chenhua Road No.3888, Songjiang, Shanghai 201602, China; ygsong@cemps.ac.cn; 3 Shanghai Chenshan Plant Science Research Center, Chinese Academy of Sciences, Chenhua Road No.3888, Songjiang, Shanghai 201602, China; 4 Natural History Museum Fribourg, Chemin du Musée 6, 1700 Fribourg, Switzerland

**Keywords:** biodiversity loss, global change, lianas, shrubs, trees, woody plant families

## Abstract

Woodiness evolved in land plants approximately 400 Mya, and very soon after this evolutionary invention, enormous terrestrial surfaces on Earth were covered by dense and luxurious forests. Forests store close to 80% of the biosphere’s biomass, and more than 60% of the global biomass is made of wood (trunks, branches and roots). Among the total number of ca. 374,000 plant species worldwide, approximately 45% (138,500) are woody species—e.g., trees, shrubs or lianas. Furthermore, among all 453 described vascular plant families, 191 are entirely woody (42%). However, recent estimations demonstrate that the woody domination of our planet was even greater before the development of human civilization: 1.4 trillion trees, comprising more than 45% of forest biomass, and 35% of forest cover disappeared during the last few thousands of years of human dominance on our planet. The decline in the woody cover of Planet Earth did not decelerate during the last few centuries or decades. Ongoing overexploitation, land use and climate change have pushed ten thousand woody species to the brink of extinction. Our review highlights the importance, origin and past triumph of woody species and summarizes the unprecedented recent decline in woody species on our planet.

## 1. The Importance of Woody Species

**Figure 1:**
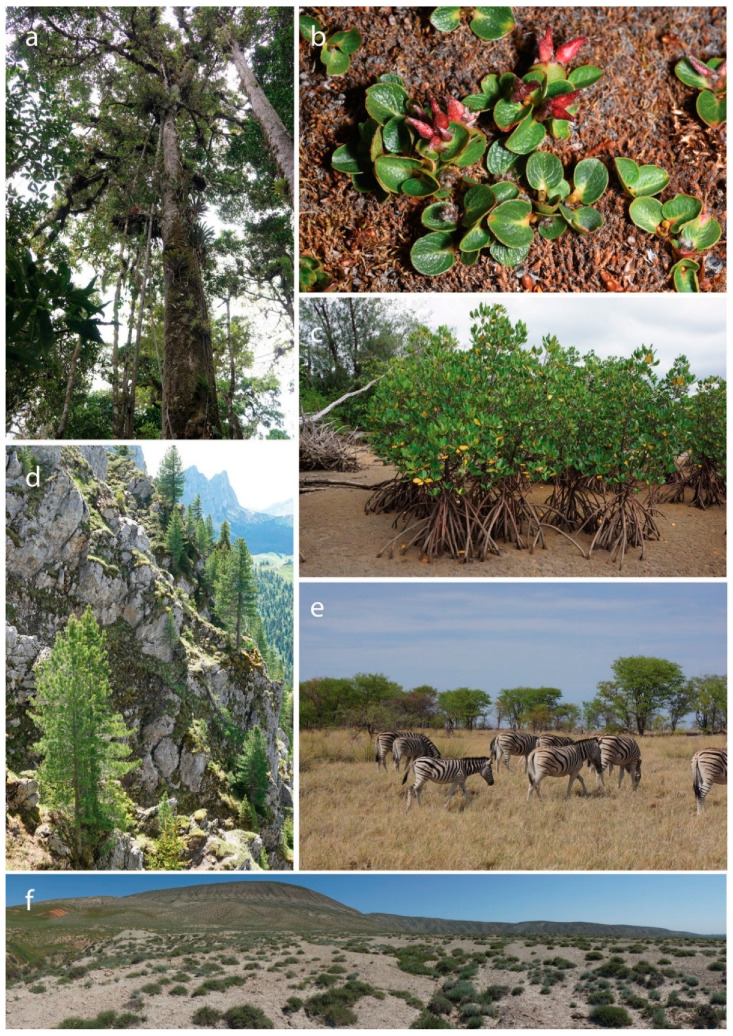
Woody plants form the principal components in forests and many other ecosystems on our planet, providing food and habitats for innumerable organisms. (**a**) *Quercus bumelioides*, tropical rainforest, Tapanti National Park, Costa Rica; (**b**) *Salix polaris*, tundra, Svalbard, Norway; (**c**) *Rhizophora stylosa*, mangrove, Iriomote, Japan; (**d**) *Pinus cembra*, alpine forest, Prealps of Fribourg, Switzerland; (**e**) Mopane woodlands, Etosha National Park, Namibia; (**f**) Semidesert vegetation, Perekushkul, Azerbaijan. Pictures: E. Kozlowski (**a**,**c**,**e**,**f**), G. Kozlowski (**b**,**d**).

Woody plants, such as trees, shrubs and lianas, form the principal components of forests and many other ecosystems on our planet [1] (Figure 1). Being among the largest and longest-living organisms, they support an immense share of the Earth’s terrestrial biodiversity, providing food and habitats for innumerable microorganisms, epiphytes and invertebrate and vertebrate species [2,3]. They have also accompanied our own species throughout its evolution: billions of people still depend on trees and shrubs for fuel, medicine, food, tools, fodder for livestock, shade, watershed maintenance and climate regulation [4]. Woody species, therefore, have inestimable scientific, economic, social, cultural and aesthetic value [5].

## 2. The Origin of Woody Plants

The earliest land plants had no vascular tissue and used ectohydric water transport by capillarity along their external surface [6]. Woodiness, which allowed endohydric, internal water transport, appeared approximately 100 million years after the conquering of land by plants [6,7,8]. The oldest wood tissue is derived from the Early Devonian, ca. 400 Mya [9]. These early woody plants were very small and used their woody structures as plumbing systems for taking up water rather than providing mechanical support [9]. Large woody plants evolved secondarily by taking advantage of the newly evolved support. Once woodiness evolved, however, the evolution of large woody trunks (so-called hyperstele) was extremely rapid [6]. In fact, it happened almost immediately, since tree habit and large trunks were already present between 385 and 390 Mya, mainly in progymnosperms [10] but also in cladoxylopsids [11]. Moreover, fossil records suggest that these organisms formed very early complex and dense forest ecosystems. These large woody organisms changed the evolutionary dynamics of terrestrial habitats, and their decaying branches, leaves and trunks significantly altered geochemical cycles all over the Earth [10,11]. Some other plant groups (e.g., arborescent lycophytes, horsetails and ferns) also evolved strategies to form tree-like organisms [6]. However, although they had some secondary xylem, their stems were determinate and short-lived, and thus, they should be denominated as “giant herbs” rather than true woody species or trees [6].

When gymnosperms, especially conifers, started to dominate our planet, they quickly left only epiphytic, aquatic or marginal niches for lycophytes and monilophytes [6]. Consequently, for more than 225 million years, the planet Earth was a true woody planet, since all seed plants were woody trees, shrubs or lianas [12]. Interestingly, the early history of angiosperms—the most diverse and dominant plant group today—also seemed to be woody [7,13,14]. The first angiosperms, however, were probably not large canopy trees but rather small shrubs or even lianas [6]. The lianous habit is potentially a driver of the evolution of vessels (one of the main novelties of angiosperms) due to the high hydraulic conductivity per unit area necessary in thin stems. Nevertheless, by the end of the Cretaceous (65 Mya), angiosperm trees, shrubs and lianas were predominant in the majority of forest ecosystems [6]. The gymnosperms, comprising a mere 1100 extant species, play nowadays an important role only in certain forest ecosystems, notably in the boreal zone of the Northern Hemisphere [15,16,17].

## 3. How Much of the Vegetal World Is Woody Today?

A recent report of the Royal Botanic Gardens in Kew (United Kingdom) estimated the total number of plant species to be ca. 380,000 worldwide [17]. Christenhusz and Byng [18] give the total number of ca. 374,000 officially described and accepted plant species, with approximately 308,000 vascular plants [18]. FritzJohn et al. [19] estimated that approximately 45% of vascular plants are woody species, and thus there might be ca. 138,500 species of trees, shrubs and lianas worldwide. According to the IUCN Global Tree Specialist Group, of this number, ca. 60,000 species are trees (43% of all woody species) [4]. The remaining 78,500 (57%) woody species are shrubs and lianas. There are no published syntheses or global data banks allowing the estimation of the proportion of lianas alone among all woody species. It is known, however, that especially in tropical forests, lianas represent ca. 35% of vascular plant diversity [20].

**Figure 2:**
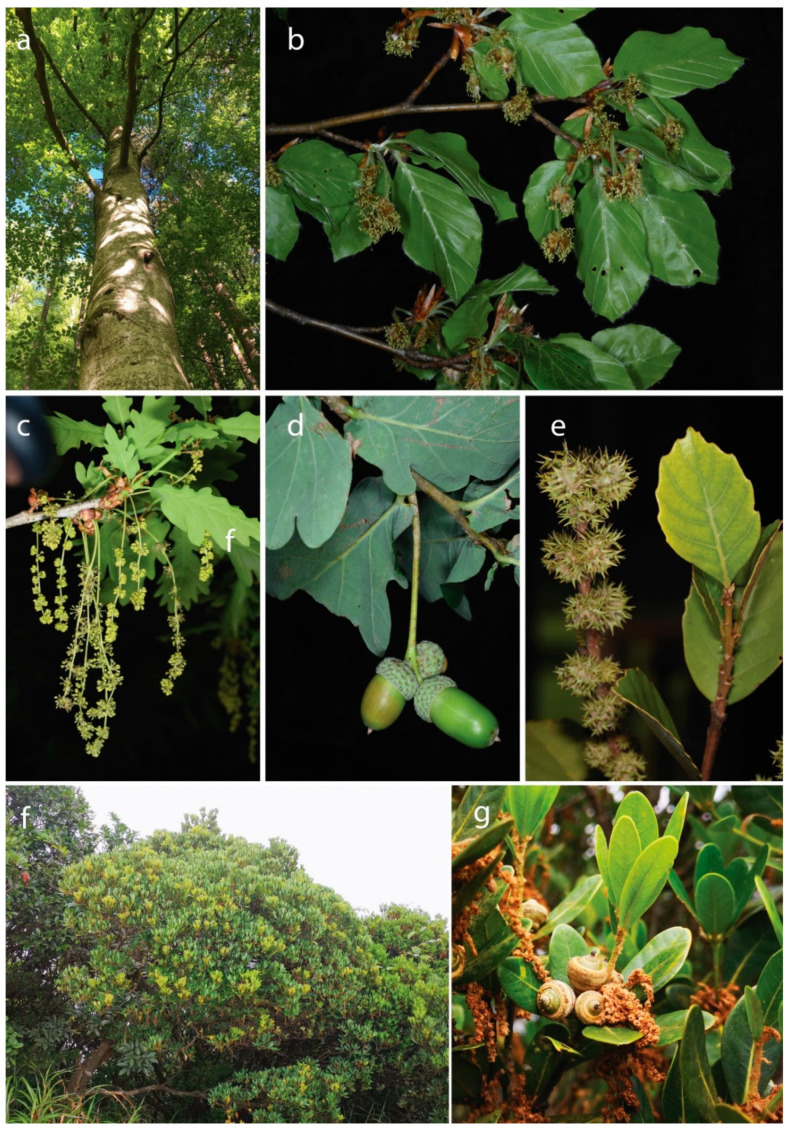
Selected members of the Fagaceae, a large and entirely woody plant family. (**a**,**b**) *Fagus sylvatica*, Ueberstorf, Switzerland; (**c**,**d**) *Quercus robur*, Enney, Switzerland. Both species belong to the most common trees in European forests; (**e**) *Castanopsis delavayi*, endemic to Southwest China. Yunnan, China; (**f**,**g**) *Quercus litseoides*, Shenzhen, Wutong Mountain, China. The species is one of the rarest and most endangered oaks worldwide. Pictures: E. Kozlowski (**a**–**e**), Y.-G. Song (**f**,**g**).

Among all 453 described vascular plant families, 191 are entirely woody (42%) (Appendix A, Figure 2). Gymnosperms are still (and as explained above, were in their long evolutionary history) clearly the woodiest plant group, with no herbaceous species at all among all known fossil and extant taxa [15,16]. The members of the other still extant ancient plant groups—e.g., lycopods and monilophytes (ferns and horsetails)—are entirely herbaceous today [19].

The overwhelming majority of trees, shrubs and lianas living today belong to angiosperms (Appendix A). Among the basal dicots, not less than 5 orders contain only woody plants (Amborellales, Austrobaileyales, Canellales, Magnoliales and Laurales) [18,21]. Many important for mankind, species-rich and entirely woody families, such as Myristicaceae (520 spp.), Magnoliaceae (294 spp.), Annonaceae (2500 spp.) and Lauraceae (2850 spp.), are basal dicots (Appendix A).

Among the eudicots, there are at least 6 entirely woody orders (Trochodendrales, Vitales, Fagales, Picramniales, Garryales and Aquifoliales). The ecologically most important, species-rich and entirely woody eudicot families belong to Proteaceae (1660 spp.), Vitaceae (910 spp.), Fagaceae (927 spp., Figure 2), Salicaceae (1220 spp.), Myrtaceae (5950 spp.), Anacardiaceae (860 spp.), Loranthaceae (1050 spp.), Sapotaceae (1273 spp.), Ebenaceae (800 spp.) and Oleaceae (790 spp.).

Additionally, several smaller woody plant families have played an important role in the historical biogeography and evolution of forests and woody flora during the past million years. These families, composed nearly exclusively of relict genera of trees and shrubs, possess enormous scientific value (for example, Altingiaceae with *Liquidambar*, Hamamelidaceae with *Parrotia* or Ulmaceae with *Zelkova*) [22]. Moreover, many of them have significant economic and cultural value, such as the Walnut family (Juglandaceae) [23]. Notably, practically all 60 species of Juglandaceae are important, useful trees for mankind. They are used for their high-quality wood, planted in parks and gardens as ornamental trees, and used for their edible fruits as well as for medicinal properties and are also used in tanning, staining, oil manufacturing and fish poisoning [24].

Interestingly, there are five taxonomic lineages across angiosperms that are not only exclusively herbaceous but that have in fact completely lost the vascular cambium during their evolution: (1) the whole order Nymphaeales, the plants of the genera (2) *Ceratophyllum* and (3) *Nelumbo*, (4) the family Podostemaceae and (5) all monocots [12]. For the first four plant groups, the convergent loss of the vascular cambium appears to be associated with the transition to an aquatic habitat [14]. Although not completely proven, this could also be the case for monocots due to the putative semiaquatic habitat of the last common ancestor of this group [25]. According to this argument, all monocot families, including those with common tree-like or shrub-like habits such as Arecaceae (2600 spp.) or Pandanaceae (982 spp.) should not be considered as woody taxa (Appendix B). According to Cronk and Forest [6], this group of plants should also be denominated as “giant herbs”.

## 4. Domination of Woody Species

The domination of woody species on our planet was demonstrated recently by Crowther et al. [2]. According to their study, there are approximately 3.04 trillion trees worldwide, which grow mainly in tropical and subtropical forests (43%) and in boreal (24%) and temperate regions (22%) [2]. Even more impressive, however, are the estimations of the biomass of woody species [26]. The overall biomass composition of the biosphere was estimated at 550 gigatons of carbon (Gt C). Plants make up the majority of the biosphere (ca. 450 Gt C), and the stems and trunks of trees (wood) represent 70% of plant biomass (ca. 315 Gt C). Thus, only trees—not including other woody species—make up approximately 60% of the total biomass of our biosphere. In comparison, all animals (and humans) taken together make up merely 2 Gt C and thus less than 0.37% of the overall biomass of our planet [26].

Furthermore, according to estimations of Reichstein and Carvalhais [27], forests (e.g., the main plant community made of woody species) store close to 80% of all the biomass on Earth. However, even in sylvipastoral landscapes and in the tundra, the proportion of the biomass of woody species is between 50 and 75% [28,29].

## 5. The Manmade Decline

Ever since *Homo sapiens* started to increase in numbers and to colonize or discover new territories or islands, regional ecosystems have been altered by human presence and activities. Notable examples are the role of *H. sapiens* in the extinction of the Pleistocene megafauna which led to modification of ecosystems at a continental scale or the arrival of humans to large islands such as New Zealand or Madagascar [30]. These human-influenced faunal extinctions are increasingly thought to have also affected plant communities at a large scale and increased biomass burning [30]. The discovery and spread of agriculture approximately 12,000–10,000 years ago led to unprecedented manmade ecosystem alterations (e.g., deforestation, domestication of plants and animals and irrigation) [30,31]. Although these human activities may have affected ecosystems on a local to continental scale, preindustrial societies lacked the necessary numbers, technology or organization to affect the environment at a global scale [31]. With the industrial development that started in ca. 1800, the global population increased more than six-fold. Deforestation and habitat losses for agricultural purposes, logging or urbanization prevailed and mankind transformed the environment at a global scale. Humans have changed the world’s ecosystem during the past 50 years more rapidly and at a larger scale than ever before [32]. These changes have placed the woody domination of our planet into peril [2].

According to Crowther et al. [2], the global number of trees has fallen by approximately 46% since the start of human civilization (disappearance of ca. 1.40 trillion of trees). Thus, humans have reduced the number of trees by approximately half. In terms of biomass, if we extrapolate based on the estimation of Bar-On et al. [26], this would indicate that approximately 40% of the total planet biomass (ca. 220 Gt C) has been lost during only the last few thousand—or perhaps even the last few hundred—years. It is difficult to imagine what effect these dramatic changes have had on global biogeochemical cycles, carbon sequestration on our planet, as well as the decreases in other organisms depending directly or indirectly on woody species.

Humans dramatically reduced not only the biomass of woody species but also their diversity (number of species). The list of recently extinct woody taxa is long and covers all taxonomic groups and all continents and biomes, and it includes for example *Nesiota elliptica* (Rhamnaceae) from Saint Helena, *Ilex gardneriana* (Aquifoliaceae) from India and *Kokia lanceolata* (Malvaceae) from Hawaii (www.redlist.org). The IUCN Red List of Threatened Species enumerates 78 trees, 49 shrubs and one liana species, which are either globally extinct (EX) or extinct in the wild (EW). However, the global assessments of all woody species are by far not complete. If we take only the trees into consideration, the Global Tree Assessment (https://globaltreeassessment.org), coordinated by the Botanic Gardens Conservation International, estimates that approximately 40% of tree species are globally threatened. This means that more than 20,000 tree species are threatened with extinction. It is highly likely that similarly high numbers of shrub and liana species are threatened.

**Figure 3:**
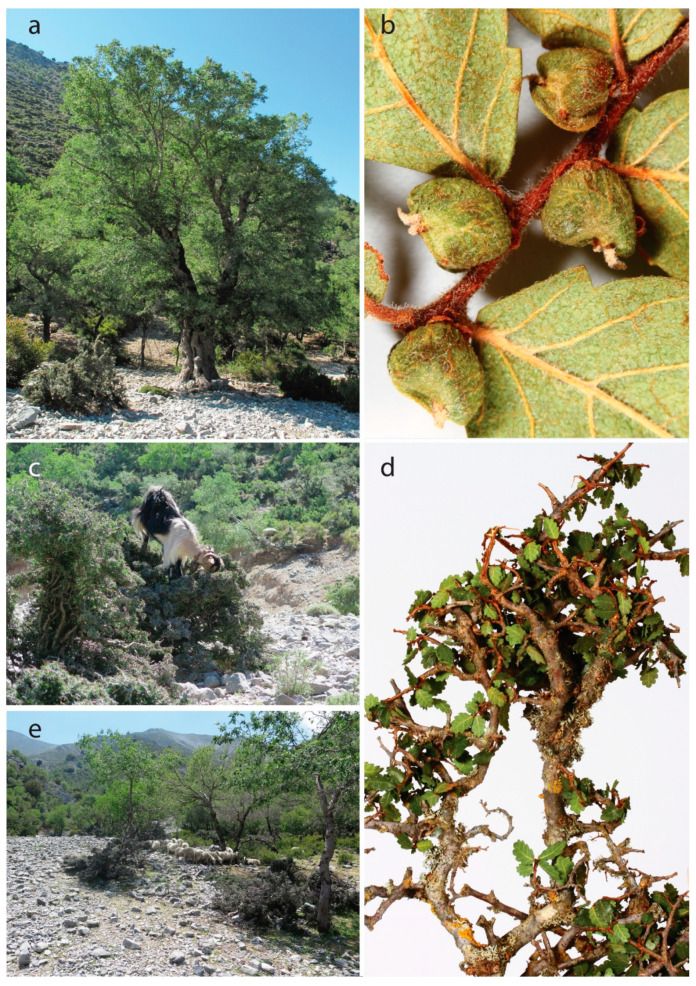
*Zelkova abelicea* (Ulmaceae), an example of an endangered tree species endemic to the Mediterranean island of Crete (Greece). Only large trees can produce fruits (**a**,**b**). However, the majority of fruit is empty, which is probably due to unfavorable climatic conditions such as pronounced and recurrent droughts. The overwhelming majority of individuals, however, are dwarfed and nonfruiting due to overbrowsing by goats (**c**,**d**). The regeneration of populations via seedlings is nearly impossible due to the overgrazing, trampling and soil erosion caused by omnipresent large caprine and ovine flocks (**e**). Pictures: G. Kozlowski (**a**), H-R Siegel (**b**,**d**), L. Fazan (**c**,**e**).

Since each of these endangered woody species is confronted with multiple threats, the elaboration of efficient conservation measures is extremely complex. For example, *Zelkova abelicea* (Ulmaceae) (Figure 3), which is a relict tree endemic to the Mediterranean island of Crete (Greece), has been classified as endangered (EN) according to the IUCN Red List (www.iucnredlist.org), and the species is mainly threatened by overbrowsing by goats [33]. There are only a few highly isolated populations composed of large fruiting trees, whereas heavily browsed dwarf individuals do not produce fruits at all [34,35]. The regeneration of populations via seedlings is nearly impossible due to the overgrazing, trampling and soil erosion caused by omnipresent large caprine and ovine flocks. Moreover, *Z. abelicea* stands are threatened by recurring droughts and wildfires as well as by ongoing climatic changes [36]. Like that of *Z. abelicea*, long-term conservation of the majority of endangered woody species will require an enormous investment of time and resources.

Recent conservation initiatives and research projects are attempting to change this dramatic loss of woody species diversity and deterioration or vanishing of the forest cover. On the one hand, there are several working groups associated with the International Union for Conservation of Nature (IUCN), which are conducting global inventories of the diversity and threats of woody species (e.g., Global Tree Assessment, https://globaltreeassessment.org), and developing solutions and specific conservation actions in order to save the World’s threatened trees (e.g., Global Tree Campaign, https://globaltrees.org). On the other hand, recent literature from forest sciences offers silvicultural management solutions for maintenance and restoration of forest cover and woody species diversity [37], including old-growth forests [38] and endangered rare species that inhabit them [39].

## 6. Conclusions

The conquest of the Earth by woody species started approximately 400 million years ago. Woody species and their communities (e.g., forests, savannas and shrub ecosystems) dominated the biosphere of our planet in terms of both biomass and diversity for hundreds of millions of years. Despite the recent dramatic biomass depletion and diversity loss of this important plant group, the Earth is still a woody planet. Trees and other woody species are the living foundation on which most terrestrial biodiversity is built [1]. To efficiently protect threatened trees, shrubs and lianas and to stop the degradation of their declining ecosystems, existing international conservation programs must be supported, and new global protection initiatives must be developed [5].

Much more must also be done at the scientific level. Local, national and international research programs must be launched, and well-coordinated working groups dedicated to specific taxonomic or geographical groups must be formed to improve our knowledge of the most threatened and least known and studied among the ten thousand endangered woody species.

## Appendix A

**Table A1:** List of vascular plant families composed entirely of woody species with an approximate number of genera and species [15,17,18,19,21,23,40]. Asterisks (*) indicate orders entirely composed of woody taxa (all species in all families are woody). The life forms are listed in order of importance. T: trees; S: shrubs; L: lianas; O: other woody forms—e.g., palm-like cycads. Capitals: the indicated life form is very common within the family. Lower case letters: the indicated life form is rarely found within the family. The order of plant groups and families follows Christenhusz and Byng [18].

Plant Group	Family	No Genera/No Species	Life Forms
**GYMNOSPERMS**			
Cycadales *	Cycadaceae	1/107	O
	Zamiaceae	9/230	O
Ginkgoales *	Ginkgoaceae	1/1	T
Welwitschiales *	Welwitschiaceae	1/1	O
Gnetales *	Gnetaceae	1/43	L, t
Ephedrales *	Ephedraceae	1/68	S, l
Pinales *	Pinaceae	11/228	T, s
Araucariales *	Araucariaceae	3/37	T
	Podocarpaceae	19/187	T, S
Cupressales *	Sciadopithyaceae	1/1	T
	Cupressaceae	29/149	T, S
	Cephalotaxaceae	1/8	T, S
	Taxaceae	5/20	T, S
**BASAL DICOTS**			
Amborellales *	Amborellaceae	1/1	T
Austrobaileyales *	Austrobaileyaceae	1/1	L
	Trimeniaceae	1/8	T, S, L
	Schisandraceae	3/85	T, S, L
Canellales *	Canellaceae	5/23	T, s
	Winteraceae	5/65	S, T, l
Magnoliales *	Myristicaceae	21/520	T, s
	Magnoliaceae	2/294	T, S
	Degeneriaceae	1/2	T
	Himantandraceae	1/2	T
	Eupomatiaceae	1/3	S, t
	Annonaceae	105/2500	T, S, L
Laurales *	Calycanthaceae	3/10	S, T
	Siparunaceae	2/75	S, T, l
	Gomortegaceae	1/1	T
	Atherospermataceae	6/16	T, S
	Hernandiaceae	5/58	T, S, L
	Monimiaceae	24/217	T, S, L
	Lauraceae	45/2850	T, S
**EUDICOTS**			
Ranunculales	Eupteleaceae	1/2	T, S
	Lardizabalaceae	7/40	L, s
Proteales	Sabiaceae	3/66	T, S, L
	Platanaceae	1/8	T
	Proteaceae	83/1660	S, T, o
Trochodendrales *	Trochodendraceae	2/2	T, S
Gunnerales	Myrothamnaceae	1/2	S
Saxifragales	Peridiscaceae	4/12	T, S
	Altingiaceae	1/15	T, S
	Hamamelidaceae	26/86	S, T
	Cercidiphyllaceae	1/2	T
	Daphniphyllaceae	1/30	T, S
	Iteaceae	2/18	T, S, l
	Grossulariaceae	1/150	S
	Aphanopetalaceae	1/2	L
	Tetracarpaeaceae	1/1	S
Vitales *	Vitaceae	14/910	L, S, T
Fabales	Quillajaceae	1/3	T
	Surianaceae	5/8	T, S
Rosales	Barbeyaceae	1/1	T
	Elaeagnaceae	3/60	S, T, l
	Ulmaceae	7/54	T, S
Fagales *	Nothofagaceae	1/43	T, S
	Fagaceae	8/927	T, s
	Myricaceae	3/57	S, t
	Juglandaceae	10/60	T, s
	Casuarinaceae	4/91	T, S
	Ticodendraceae	1/1	T
	Betulaceae	6/167	T, S
Cucurbitales	Anisophylleaceae	4/71	T, S
	Corynocarpaceae	1/5	T, S
	Tetramelaceae	2/2	T
Celastrales	Lepidobotryaceae	2/2	T
Oxalidales	Huaceae	2/4	T, S
	Connaraceae	12/180	T, S, L
	Cunoniaceae	27/330	T, S
	Elaeocarpaceae	12/615	T, S
	Brunelliaceae	1/60	T
Malpighiales	Pandaceae	3/17	T, S
	Irvingiaceae	3/13	T
	Ctenolophonaceae	1/2	T
	Rhizophoraceae	15/147	T, S
	Erythroxylaceae	4/242	S, T
	Bonnetiaceae	3/35	T, S
	Clusiaceae	13/750	S, T
	Calophyllaceae	14/475	T
	Caryocaraceae	2/26	T, S
	Lophopyxidaceae	1/1	S
	Putranjivaceae	2/216	T, S
	Centroplacaceae	2/6	T
	Balanopaceae	1/9	T, S
	Trigoniaceae	5/28	T, S, l
	Dichapetalaceae	3/170	T, S, L
	Chrysobalanaceae	18/533	T, S
	Humiriaceae	8/56	T, S
	Goupiaceae	1/2	T
	Lacistemataceae	2/14	S, T
	Salicaceae	56/1220	S, T
	Ixonanthaceae	3/17	T, S
	Picrodendraceae	25/96	T, S
Myrtales	Combretaceae	10/530	T, S, L
	Vochysiaceae	7/217	S, T
	Myrtaceae	132/5950	T, S
	Crypteroniaceae	3/13	T
	Alzateaceae	1/1	T, s
	Penaeaceae	9/32	S, T
Crossosomatales	Aphloiaceae	1/1	S, t
	Geissolomataceae	1/1	S
	Strasburgeriaceae	2/2	T
	Staphyleaceae	2/45	T, S
	Guamatelaceae	1/1	S
	Stachyuraceae	1/8	S, T, l
	Crossosomataceae	4/10	S, t
Picramniales *	Picramniaceae	3/49	T, S
Sapindales	Kirkiaceae	1/6	S, T
	Burseraceae	19/615	T, S
	Anacardiacaeae	83/860	T, S, L
	Simaroubaceae	22/108	T, S
	Meliaceae	53/600	T, S
Huerteales	Petenaeaceae	1/1	T, s
	Gerrardinaceae	1/2	S, t
	Tapisciaceae	2/6	T
	Dipentodontaceae	2/20	T, S
Malvales	Muntingiaceae	3/3	T, S
	Sphaerosepalaceae	2/18	T, S
	Sarcolaenaceae	10/71	T, S
	Dipterocarpaceae	16/695	T
Brassicales	Akaniaceae	2/2	T
	Caricaceae	6/35	T, S
	Setchellanthaceae	1/1	S
	Koeberliniaceae	1/2	S, T
	Bataceae	1/2	S
	Salvadoraceae	3/11	S, T
	Tiganophytaceae	1/1	S
	Pentadiplandraceae	1/1	S, l
	Capparaceae	30/324	S, T
Berberidopsidales	Aextoxicaceae	1/1	T
	Berberidopsidaceae	2/3	L
Santalales	Olacaceae	29/180	T, S, L
	Opiliaceae	11/33	T, S, l
	Misodendraceae	1/8	S
	Loranthaceae	76/1050	S, t, l
Caryophyllales	Tamaricaceae	4/78	S, T
	Dioncophyllaceae	3/3	S, L
	Ancistrocladaceae	1/21	L, S
	Rhabdodendraceae	1/3	S, T
	Simmondsiaceae	1/1	S
	Physenaceae	1/2	S, T
	Asteropeiaceae	1/8	T, S
	Achatocarpaceae	2/11	S, T
	Stegnospermataceae	1/4	T, S
	Barbeuiaceae	1/1	L
	Sarcobataceae	1/2	S
	Didiereaceae	7/22	T, S, l
Cornales	Nyssaceae	5/37	T
	Curtisiaceae	1/1	T
Ericales	Marcgraviaceae	7/120	L, S, t
	Tetrameristaceae	3/5	T, S
	Fouquieriaceae	1/11	S, T
	Lecythidaceae	25/355	T, s
	Sladeniaceae	2/3	T
	Pentaphylacaceae	12/330	T, S
	Sapotaceae	54/1273	T, S, L
	Ebenaceae	4/800	S, T
	Theaceae	9/240	T, S
	Symplocaceae	2/260	T, S
	Styracaceae	11/160	T, S
	Roridulaceae	1/2	S
	Actinidiaceae	3/360	L, T, S
	Clethraceae	2/75	S, T
	Cyrillaceae	2/2	S, T
Icacinales	Oncothecaceae	1/2	T, S
	Icacinaceae	25/165	T, S, L
Metteniusales	Metteniusaceae	11/50	T, S, l
Garryales *	Eucommiaceae	1/1	T
	Garryaceae	2/25	T, S
Gentianales	Gelsemiaceae	3/11	S, T, L
Solanales	Montiniaceae	3/5	S, T
Lamiales	Plocospermataceae	1/1	S, T
	Oleaceae	26/790	S, T, L
	Schlegeliaceae	4/37	T, S, L
	Thomandersiaceae	1/6	S, T
	Paulowniaceae	3/8	T, L
Aquifoliales *	Stemonuraceae	12/90	S, T
	Cardiopteridaceae	5/43	T, S, l
	Phyllonomaceae	1/4	T, S
	Helwingiaceae	1/4	S, t
	Aquifoliaceae	1/500	T, S
Asterales	Rousseaceae	4/6	S, T, l
	Alseuosmiaceae	5/13	S
	Phellinaceae	1/12	S, T
	Argophyllaceae	2/21	S, T
Bruniales	Bruniaceae	6/81	S, t
Paracryphiales	Paracryphiaceae	3/36	S, T
Apiales	Pennantiaceae	1/4	S, T, l
	Torricelliaceae	3/10	T, S
	Griselinaceae	1/7	S, L, T
	Pittosporaceae	7/245	T, S, L
	Myodocarpaceae	2/15	T, S

## Appendix B

**Table A2:** List of monocot plant families composed entirely of tree-, shrub- and liana-like organisms [21]. Monocots have completely lost the vascular cambium during their evolution and thus should be denominated as “giant herbs” rather than true woody species [6]; therefore, were not included in the Appendix A. The life forms are listed in order of importance. T: trees; S: shrubs; L: lianas (capitals: often within the family; lower case letters: rarely). The order of plant families follows Christenhusz and Byng [18].

Plant Group	Family	No Genera/No Species	Life Forms
MONOCOTS			
Pandanales	Pandanaceae	5/982	T, S, L
Liliales	Philesiaceae	2/2	S, L
	Ripogonaceae	1/6	S, L
Arecales	Dasypogogonaceae	4/16	S, t
	Arecaceae	181/2600	T, s
